# The Alchemy of Coaching: Psychological Capital as HERO within Coaches’ Selves

**DOI:** 10.3390/ijerph191912020

**Published:** 2022-09-23

**Authors:** Nur Aimi Nasuha Burhanuddin, Nor Aniza Ahmad, Rozita Radhiah Said, Soaib Asimiran

**Affiliations:** 1 Department of Foundations of Education, Faculty of Educational Studies, Universiti Putra Malaysia, Serdang 43400, Malaysia; 2 Department of Language and Humanities Education, Faculty of Educational Studies, Universiti Putra Malaysia, Serdang 43400, Malaysia

**Keywords:** positive psychology, psychological capital, instructional coaching, positive psychological resources, phenomenology

## Abstract

The complex nature of coaching challenges instructional coaches (ICs) professionally as it requires them to deal with not only teachers’ resistance, acceptance and expectation but also adhere to the complex and multifaceted roles that they are bearing. Psychological capital (PsyCap) has been upheld as an effective construct for defending against stress, negative emotions and burnout among educators. This phenomenological study explores ways in which PsyCap was experienced by Malaysian instructional coaches (ICs). Data were gathered from face-to-face interviews with seven instructional coaches purposefully selected from six different District Education Offices (DEOs) throughout Malaysia. Extracted from participants’ own words and through the exploration performed, PsyCap was experienced by the participants through a sense of responsibility, positive resources and work commitment. This study highlights the importance of PsyCap as inner positive psychological resources that aided instructional coaches in their practice of coaching. In addition, this study suggests future research recommendations towards implementing PsyCap developmental training with another group of instructional coaches.

## 1. Introduction

Following the positive movement initiated by [1] Martin Seligman (1998), coaching is shown to be parallel with the mission of positive psychology, that is, to look into ways to leverage human strengths. Broadly considered, the aim of coaching focuses on enhancement of performance, development and well-being [2]. Coaching started in the business setting and results from the studies performed were mainly associated with positive outcomes. By some extraordinary alchemy, a number of studies have illustrated positive impact of coaching when implemented in schools. For instance, a randomised controlled trial conducted with Australian high school teachers by [3] found that coaching increased goal attainment, enhanced workplace well-being and resilience, reduced stress and improved leadership styles. Another work by [4] also reported positive impact of coaching to teachers as it increases teachers’ well-being and daily practice as well as teachers’ collaboration at schools. 

However, despite the reported generally positive impacts of coaching, instructional coaching has its own sets of challenges. Instructional coaches (ICs), specifically those who bear multifaceted roles in their profession, face challenges on daily basis. The complex nature of coaching challenges ICs professionally as it requires them to deal not only with teachers’ resistance, acceptance and expectations, but also to adhere to the complex roles that they are bearing. Studies revealed that ICs are expected to adhere to three different roles that are implementer, advocate and educator [5,6,7,8,9,10]. This means that coaches have to engage in the roles depending on the context of the coaching situation and that different strategies need to be employed based on the consideration of the role they chose. Role-shifting constantly occurs depending on the situation [10]. For instance, teachers’ stages of learning are different from one individual to another in terms of their ability to reflect and make improvements to their teaching, and because of this, coaches need to adhere to multiple roles to make sure the teaching quality is enhanced [10]. Besides the multiple roles ICs need to adhere to, they also face many challenges while working with adult learners to improve their performance. [11] stated that one remarkable challenge ICs face when working with teachers is to establish a partnership with those who are reluctant to change. Teachers are less likely to give cooperation and positive feedback when they view the new instructional practices as difficult to implement and no different than the previous effort to reform classroom instruction. In addition to teachers’ resistance to receiving coaching, another challenge that ICs face is when teachers misunderstand their roles as supervisors or quasi-administrators [12,13]. Knowing this, administrators, as well as ICs, need to be clear of a coach’s role as they should not judge or evaluate teachers’ classroom practices. These findings may suggest an explanation as to why coaches experience resistance from teachers [14]. On top of that, some cases reported that coaches’ roles and responsibilities were poorly defined, and this resulted in them having to perform quasi-administrative or clerical work rather than the actual job which is to improve teachers’ performance [13] The other challenge that ICs often face is the issue of confidentiality. Teachers are more likely to work together with the ICs if they believe that the discussion is kept confidential and they are more likely to implement new practices introduced to them when they can work together in partnership with the coaches [11]. Knowing the complex nature of an IC’s duty suggests the importance of PsyCap to them as the HERO within that could contribute to the success of instructional coaching

PsyCap that stems from the discipline of positive organisational behaviour (POB) is concerned with people’s strengths rather than their weaknesses and dysfunctions and how they can grow and thrive rather than be fixed and maintained. This turns out to be a powerful asset in terms of facing notable challenges as instructional coaches (ICs). Knowing that instructional coaching offers a worthwhile but challenging experience as there will be roadblocks, interruptions and detours, it is then crucial for ICs to build and expand their positive psychological resources of PsyCap. A bulk of PsyCap research has been performed focusing on industrial organisational contexts and to this date, the study within the educational context remains scarce [15,16,17,18,19,20]. There are strong theoretical reasons to propose that PsyCap could also play a key role in the educational context. The nature of academic tasks is no different from work duties. Moreover, the characteristics that would enable academics to succeed in their organisations are quite similar to the characteristics that employees need to succeed in their organisations [21,22]. For example, in both academic and industrial contexts, a strong sense of efficacy is needed in oneself to accomplish goals [23]. Similarly, individual needs to be resilient because challenges and difficulties are bound to happen in both contexts and being able to return stronger after hardship is a good predictor of success. Educational organisations, similar to other organisations, encounter hardship, reforms, increasing standards and accountability, and these put pressure on educators [24,25]. 

## 2. Literature Review 

### Psychological Capital

Psychological capital (PsyCap) is defined as an individual’s positive psychological state of development and is characterized by **HERO**: (1) persevering toward goals and, when necessary, able to redirect paths to goal (**H**ope) to succeed; (2) having confidence (**E**fficacy) to take on and put in the necessary effort to succeed at challenging tasks; (3) when beset by problems and adversity, sustaining and bouncing back and even beyond (**R**esilience) to attain success; (4) making a positive attribution (**O**ptimism) about succeeding now and in the future [26]. It is a concept that goes beyond human capital (what you know), social capital (who you know) and financial capital (what you have). PsyCap is viewed as “who you are” and “who you are becoming”. It covers four malleable and manageable constructs: hope, resilience, efficacy and optimism [27]. Hope, efficacy, resilience, and optimism (HERO)—an acronym for PsyCap’s core constructs—is believed to have the potential to be managed and developed for one individual’s optimal flourishing. When these four travel together and interact synergistically, they produce differentiated manifestations over time and across contexts [15]. PsyCap has been linked with various positive outcomes including one’s engagement, satisfaction, motivation, happiness and well-being [28,29,30]. Psychological resources can provide the necessary energy from within the individual self to meet the demands of a changing environment [31]. PsyCap as the psychological resources that are built up by a positive mental and emotional state suggests the need for development and investment, especially within the educational society following increasing job demand. With the substantial evidence showing numerous positive outcomes of having PsyCap, refs. [32,33,34,35,36] developing and expanding one’s PsyCap is considered important.

Therefore, researching PsyCap through understanding the experience of individuals who are involved directly in coaching is an important next step to understanding the relevance of PsyCap as an inner positive psychological resource to help them face the demands of instructional coaching practices. In addition, having this information, professionals may assist ICs to further cultivate, strengthen and maintain PsyCap in any circumstances they encounter. It is believed that by developing PsyCap, organisations may be able to develop their ability to brave hardship and crises that arise. This study aimed to explore the ways in which PsyCap was experienced by Malaysian instructional coaches (ICs). A phenomenological approach was employed as it was thought to be the most appropriate approach to understand the essence of ICs’ PsyCap experience. 

## 3. Methodology

### 3.1. Research Design, Sampling, Data Collection and Ethical Considerations

An interpretative phenomenological analysis (IPA) approach was used in the study to understand the participants’ subjective experience, perception and understanding of a given phenomenon. IPA is informed by three key areas in the philosophy of knowledge, which are phenomenology, hermeneutics and idiography [37]. As the intention of this study was to explore and understand in a great extent PsyCap as experienced by instructional coaches (ICs), the phenomenological foundation of this study “aims at attaining a profound understanding of the nature or meaning of our daily experiences [38] and due to that, exploration of this phenomenon was performed with seven ICs from several different District Education Offices in Malaysia without prior knowledge of and pre-assumption about their level of PsyCap. Taking into account the second key feature of an IPA study, which is hermeneutic, understanding is drawn from a participant’s perspective, but always involves interpretation by the researcher. Informed by the hermeneutics approach of IPA, throughout this study, the researchers’ understanding of ICs’ experience of PsyCap came from the researchers’ previous understanding of PsyCap and the interpretation of what the researchers saw, heard and were told by the participants of the study. Through the researchers’ best efforts, they tried to understand the ICs’ experience of PsyCap as best as they could. This was performed through in-depth interviews that were carried out with the participants. As the researcher engages in a reflective interpretation of the text, it results in a fuller, more meaningful understanding and hence brings before him or her “something that otherwise happens ‘behind his or her back’” [39].

The justification for employing IPA in this study can be explained mainly in two ways. First, there is a lack of empirical studies in understanding how an individual experience the PsyCap of hope, self-efficacy, resilience and optimism. The abundance of previous studies using pre-existing PsyCap instruments indicated that no in-depth data were gathered, and the data gathered were potentially biased as the studies used a self-report measure. Additionally, most of the previously conducted studies researched PsyCap as the mediator. This suggests the need for more qualitative studies to be conducted to understand PsyCap in greater detail, that is, how individuals experience it and how it operates. As [40] mentioned, “Language is the means through which we gain an understanding of the world that we inhabit.” It is speech, and especially conversation, that is at the heart of all understanding. An explanation of how individuals experience the phenomenon of PsyCap needs to be developed. Without a qualitative paradigm, the research would not be able to capture such knowledge. As the intention of this study was to explore and understand to a great extent PsyCap as experienced by ICs, IPA was considered the best means for this type of study. 

Due to the aim of gathering detailed data and an in-depth understanding of each of the participants’ shared experiences, and after considering the researchers’ time and resources, only seven ICs were chosen as participants for this study. The participants of this study were chosen based on several inclusion criteria set ahead of time by the researchers. Selection for inclusion involved purposive homogenous sampling aiming to gain the most richness and depth from the data of participants who lived the experiences of the phenomenon being studied, PsyCap [41]. The participants were selected based on these criteria: the research participants had experienced the phenomenon, were immensely interested in the meaning of the phenomenon being studied, were willing to participate in a considerably long interview (or series of interviews), and allowed the researcher to record the interview and publish the data gathered [42]. We were interested in exemplary individuals who are/were successful in their practice. For the purpose of this study, “successful” was defined as ICs who were respected, capable, at the highest grade for a government officer within the educational field and were an instructional coach for more than five years. All the participants were selected for this study because of the high-performance grades they attained throughout their profession as well as their experience in educational organisations. They were unique and different from other ICs, hence the reason they were selected as the participants for the study. All of them were aged between 49 and 59 years old. They were from the same racial background and could be broadly classified as Malay. The recruitment started with the researchers gathering information on ICs that fulfilled the criteria set for this study from the School Management Division at the Ministry of Education Malaysia and the District Education Office. After obtaining the information of all ICs serving in several District Education Offices in Malaysia, only those who matched the criteria were chosen for this study. There are three reasons why such a strategy was used in selecting the participants; (1) it is not possible to include all ICs in Malaysia in the study due to their high number, (2) limitation with researchers’ time and resources to interview all of them, (3) researchers believed that ICs with the set criteria could give rich information about the phenomenon. Table 1 provides participants’ characteristics and demographics. Each participant was given a pseudonym to ensure confidentiality. The detailed sampling and recruitment strategy are outlined in Table 2 below. 

Permission to conduct this study was requested from the University Ethics Committee for Research Involving Human Subjects and the State Education Department as well as the Educational Planning and Research Division. This procedure was performed before introducing the study to the ICs and conducting data collection to ensure that these respective organisations were well informed about the research and any potential risks and harm during the study. In addition to that, this procedure was performed to ensure that the privacy of the participants was protected. Before the interview session, participants were given an information sheet that explained the details of the study as well as a consent form indicating their willingness to participate in the study. In this way, the participants were well aware of their rights while participating in the study. 

The researchers’ ontological positions and epistemological assumptions suggested that the best means to generate data on people’s experiences, understandings, views and interpretations was to interact with people, talking and listening to them through an informal conversational interview. The use of questionnaire was considered not suitable in exploring one’s experience as it is subjective and there are no absolute single answers to every individual’s experience. Experience is complex, and therefore the use of questionnaires was far from meeting the objective of the study. The researchers instead developed an interview schedule, with the content validated by the experts in the field. As the aim of the interview was to enter the participants’ lifeworlds, the interview schedule was developed using guidelines described by [43]. In the present study, the researchers prepared 10 questions along with the possible prompts as suggested by [37] for IPA study. The construction of the interview schedule took into consideration key issues, both conceptual and theoretical. This interview schedule consisted of open questions in order to encourage expansive answers rather than yes/no answers from participants. The content included reflection on the ICs’ professional life as well as personal life, reflection on the challenges and difficulties they faced and good and bad events that they encountered throughout their period of being an IC. The participants were also asked to reflect on how they dealt with every event in their life, both good and bad. The questions were funnelled from the most general to the most specific. This allowed the participants to focus on his/her concerns rather than the researchers’ [44]. The researcher also made sure that the questions developed were clear and simple, avoiding technical language so that participants could easily understand. 

All interview data were held in a secure location and tape-recorded interviews were each assigned an identity code to ensure the confidentiality of all participants. The participants were assured that all research, writing and publication would be anonymous. As such, pseudonyms were used instead of their real names and anticipated benefits and potential hazards were also explained to the participants. Addressing ethical issues before conducting an interview is crucial because an in-depth interview may have unanticipated long-term effects on the participants [45]. The questions to be asked during the interview were carefully constructed by weighing both risks and benefits they might bring to the informants. As stated by [46], “an interviewer’s task is first and foremost to gather data, not change people. Interviewers also need to be responsive to the human issue, including great suffering and pain that may unfold during an interview” (p. 354). The researcher addressed all these ethical issues during the period of the study. As [47] stated, “Qualitative researchers are guests in the private spaces of the world. Their manners should be good and their code of ethics strict” (p. 459).

Each of the interview sessions with the participants lasted for 60-90 min. Whole conversations were transcribed by the researchers, including nonverbal cues, extraneous words and utterances such as “hmm”. This was performed to ensure that no data were left out and it allowed the researchers to focus on and be fully immersed in the data through a continuous cycle of listening, reading and reflecting on the data. To ensure validity, the transcribed interview copy was given to the participants for their verification. The interview session began by first building rapport with the participants. This created a friendly environment so that the participants felt comfortable. The session began with small talk, after which the researchers slowly asked the participants the actual interview questions and tried keeping the conversation as smooth as possible, in a casual manner but with a direction. Whenever the participants went beyond the focus of the study and shared things unrelated to the research concern, the researchers pulled them back to the research topic. During the interview, a probing technique was used to clarify some thoughts and uncertainty. Additionally, “ok” or “mm” or just silence were used as an indication that participants could continue with their sharing. During the interview, the researchers followed a free discourse format that allowed the participants to talk as much as they wanted. However, the researchers were cautious in order to ensure that the participants were in control during the interview process.

### 3.2. Data Analysis

The process of data analysis was performed simultaneously with the process of data collection. This was so for several reasons. Firstly, to ensure that a more suitable probing question could be asked during the next scheduled interview. Secondly, to ensure that no important data were left out by the researchers: This was because had the researchers waited until the last interview with the participants before transcribing and analysing the data, they could have unintentionally forgotten details that could be significant to the research. Hence, once each interview was conducted, the researchers began transcribing the interview audio and also at the same time analysing the transcript. All of the interviews were transcribed word to word by the researchers. The researchers put in effort to make sense of the data collected before proceeding to finding codes and themes. [48] suggested that researchers should “read the transcripts in their entirety several times. Immerse themselves in the details, trying to get a sense of the interview as a whole before breaking it into parts” (p. 103). This phenomenological study used a method of analysis that is beyond the generic approach to data analysis. This study followed the step-by-step, unidirectional guideline for data analysis proposed by Yardley [37] starting from (1) reading and re-reading, leading to (2) initial noting, (3) developing emergent themes, (4) searching for the connections across emergent themes, (5) moving to the next case and (6) looking for patterns across cases.

### 3.3. Validity and Reliability

In this present study, validity and reliability of the study findings were ensured by following Yardley’s principles of assessing the quality of qualitative research [49]; (1) sensitivity to context, (2) commitment and rigour, (3) transparency and coherence and (4) impact and importance. In the present study, the researchers made the effort to be sensitive to the context in several ways. Firstly, a literature review related to the topic of the study was performed to describe and support the rationale for the choice of methodology, data collection and analysis procedure. Secondly, the researchers continuously reflected on sociocultural influences such as language, culture and beliefs to understand how these factors could impact their approach to the research, data collection, data analysis and finally, the presentation of the findings. This reflection included working through the research details with the research members, writing field notes at the point of data collection and analysis, and thinking about how certain things should be performed differently to obtain much better data, with great detail and in-depth understanding. Finally, the researchers were sensitive with regard to their relationship with the participants, especially in making the participants feel comfortable with their presence.

## 4. Findings

The narratives of the participants were interpreted by highlighting the similarities and differences between the participants’ stories. The participants’ experiences of PsyCap and the researchers’ interpretations of the experiences were developed into a conceptual understanding and meaning [37]. Themes on how participants experienced PsyCap of hope, efficacy, resilience and optimism (HERO) can be explained through three super-ordinate themes: “a sense of responsibility”, “positive resources” and “work commitment”. The themes identified were based both on their relevance and commonality within the transcripts and on individual accounts that captured a unique in-depth perspective [37]. As indicated in Table 3, the super-ordinate themes are presented, with sub-ordinate themes nested within them. 

### 4.1. A Sense of Responsibility

Prior to applying for the IC position, all the participants had aspirations about the changes they wanted to bring to the Malaysian education system, as they viewed it as their responsibility. They wanted to reform the current education system, with the hope that they could contribute to resolving issues that were stagnant for a long time. They viewed IC as an opportunity to effect the changes that they wanted to introduce but had no chance of before. All the participants felt that they were responsible for the future generation’s educational success. They believed that with their personal qualities, they could help teachers at school perform their tasks better, and further contribute to the school’s success.

#### 4.1.1. Responsibility to Others

The participants expressed their experience of PsyCap concerning their feelings of responsibility toward others, and thus, PsyCap acted as the internal psychological resources that helped the participants perform their tasks as an IC. In this way, the participants conveyed the feeling that they were responsible as the “agent of change” in the effort of reforming the current educational system. They stepped out of their comfort zone and leapt from their former teaching profession to become ICs. Ahmad said in the interview,

“I stepped down and left my previous position as a lecturer. When I was a lecturer, I saw there was a need for me to make a change... so I left my career as a lecturer”

(Ahmad)

Another participant, Sheila, expressed feeling responsible when she always had lingering questions in her mind about whether the teachers she guided previously (when she was a lecturer in the Teacher Training Institute) were able to perform the tasks as a teacher well enough. She displayed her concern by saying,

“During my time at the Teacher Training Institute, I have always wondered, once they have finished their training at Teacher Training Institute, can they practice what they have learnt?”

(Sheila)

The other participants expressed their experience of PsyCap through their feeling of “giving back to others”. Some of the participants were posted as a teacher outside of their hometowns. For years, they had been dreaming of returning and serving the people in their hometown and sharing with the teachers and students the knowledge that they acquired throughout their profession as Excellent Teachers. Asyikin recalled,

“My only aim is to make my state successful. I came back serving my state so I can contribute back and guide the teachers to be at par with those at the international level”

(Asyikin)

Helping teachers at school was one of her main reasons to come back to her hometown, Terengganu, and it was the experiences she acquired that motivated her to come back. She felt the need to bring about change to the education system, precisely in her hometown. She had the confidence that her expertise could benefit others. 

#### 4.1.2. Realising the Skills and Quality in Self

Participants also expressed that realising the skills and qualities they have was part of their experience of PsyCap. Participants realised that to create change in others, which is considered at a big scale, it is also important for them to first acknowledge the skills and qualities that they have within themselves. Having self-confidence would then become the pushing factor for them to coach others. Sheila said,

“When the ministry first announced this IC position and knowing the role and task it had to do which is to guide teachers, I saw the potential in me, that I believe I have the criteria as well as the knowledge that I can share with the teachers”

(Sheila)

This was similar to Ahmad, who believed that he had confidence in his skills; he said,

“I have the ability to know people because I am very experienced and have been involved in teaching and training for a long time”

(Ahmad)

### 4.2. Positive Resources

Participants described their experience with PsyCap as positive resources that enable them to constantly think positively about things that happened. It has been established in the literature that ICs also face challenges while they are doing their job, for instance, reluctance from teachers and ambiguous job roles. Participants emphasised that PsyCap helped them maintain a positive mind, attitude and behaviour.

#### 4.2.1. Keeping a Positive Mind

The participants shared during the interview how the tasks of an IC could sometimes be challenging. They shared that PsyCap was experienced as positive resources that helped them in doing their tasks as ICs. Despite the challenges they faced in the practice of instructional coaching, the participants always looked on the bright side. Ahmad, for instance, said,

“Challenges are the foundation of our success. There is no success without challenges. The challenges make us think. We can never be successful if we are too comfortable”

(Ahmad)

Similarly, Sheila discussed turning the difficulty into something motivating for her. She said,

“The tasks we need to do keep changing and this becomes a motivation for me to keep being an IC”

(Sheila)

Asyikin’s experience with PsyCap in the practice of instructional coaching could be displayed through how PsyCap managed to keep her positive whenever something bad happened. According to her, when such unfavourable circumstances happened, she said,

“I have always put aside negative elements. I told myself, ‘Not me, not me, not me. It’s not me! I’m a very positive person’”

(Asyikin)

The conversation that the researchers had with Asyikin signified that PsyCap was experienced as personal resources that not only contributed to her positive thinking, but also influenced how she interpreted things that happened in her life. For Zaiton, despite being an expert in the field of mathematics, her journey as an IC was not an easy one. She faced several hardships along the way in her career as IC. She was under so much pressure at the time, as teachers did not accept her presence at school. The teachers rejected her presence at school openly via verbal and facial expressions. They also spoke to her in a disrespectful way and some even threw harsh words at her. She recalled that she had to take medication as she was then diagnosed with hypertension.

“It gave me so much pressure that I was prescribed hypertension medication. I have never had hypertension before!”

(Zaiton)

Following this unfavourable occasion with teachers she had to coach, she also mentioned that there were several other negative occasions that she faced during her career as an IC. However, despite all the adversities she faced in her career, she then said,

“In the end, it (the heated discussion with teachers) turned out to be a good event. One thing is probably because I am much older than them. It’s also because I never forced them to follow my way of doing, and I never handle things negatively”

(Zaiton)

She then remarked how being older than the teachers gave her some advantage in coaching. She attributed the negative events as temporary and detailed how she never reacted negatively, despite unfavourable events with reluctant teachers.

#### 4.2.2. Positive Attitude and Behaviour

Participants also discussed their experience of PsyCap that related to positive attitude and behaviour. Zaiton illustrated how she approached negative events through positive attitude and behaviour. When unpleasant events occurred between her and the teachers, she decided to react positively and calmly to the situation instead of adding fuel to the fire. In one event when there was a conflict between her and some of the teachers she coached, she recalled,

“Since they have been throwing bad words at me, I decided to change the strategy of approaching them. What I did was, I said to them, ‘Alright, since you are so mad having me here to coach you, go on and say what you wanted to say first’. I used that kind of approach and I’m cool with that”

(Zaiton)

Having PsyCap keep her composed and reacting positively no matter how tough the situation was. This is similar to Yahya, who shared that he never forced his teachers to follow his suggestions. He would guide them, but never forced them to listen to him. He stated that he understood andragogy and that it helped him tremendously in dealing with teachers. He said in the interview,

“Even our SOP highlighted that we (as IC) cannot force teachers who do not want to be coached. That was the concept. We cannot force them”

(Yahya)

PsyCap here, as told and experienced by Yahya, regulated his action and behaviour in dealing with teachers during the practice of instructional coaching. While Yahya discussed the importance of understanding teachers, Asyikin shared her experience of PsyCap concerning one’s personality. She stressed how vital it is for coaches to internalise and exercise humanistic values within them. According to her,

“Our personality, we need to be humble. We want to coach people, of course we need to have humanistic characteristics”

(Asyikin)

In addition to humanistic values being instilled within the coach, Ahmad pointed out the importance of being honest. To him, having a positive attitude by being honest makes a lot of difference in instructional coaching. He said,

“We, as IC need to be honest. We need to gain their trust or else. And once we lose their trust, they are never going to believe us”

(Ahmad)

### 4.3. Work Commitment

Many participants spoke about their experience with PsyCap concerning their work commitments. They seemed to focus on the presence of PsyCap by associating it with their commitment to working and pushing themselves to give the best they could to help teachers in their instructional skills. 

#### 4.3.1. Knowledge-Seeking Behaviour

The participants’ experience of PsyCap was often discussed as linked to knowledge-seeking behaviour, in which participants continuously improved themselves through knowledge to make sure they were well-prepared to coach teachers. For most participants, having the right knowledge facilitated the process of instructional coaching as it not only built their confidence but also the teachers’ confidence in them. Ahmad said,

“To be a successful coach, I have to know the principles of counselling. I have to learn, I have to read”

(Ahmad)

Realising their knowledge of coaching was inadequate, the ICs took the initiative to equip themselves with the skills and knowledge needed to perform the task as ICs better, instead of waiting for the ministry to provide proper training. Siti said, “Even though we as IC were never given an exposure (to coaching), we still need to study and do our homework” (Siti). 

For Asyikin, taking coaching courses also helped her become more confident in her job. She said, “Since I joined the neuro linguistic programming (NLP), I felt much more confident to coach people.” (Asyikin).

Similarly, Yahya, who realised he needed to equip himself with proper knowledge, said in the interview, “At the same time, we accept what we are lacking in and acknowledge that we seek knowledge and take relevant courses. As for me, I am currently taking a course at a teacher training institute” (Yahya).

#### 4.3.2. Have Goals Set in Mind and Work Hard in Achieving Those Goals

The participants’ experience of PsyCap was also demonstrated when they shared the goals they wanted to achieve while being an IC. The participants had goals set in mind and were working hard to achieve those goals. Laila stated, “I have my objectives and am working hard towards achieving those objectives” (Laila). 

Siti also shared her experience with PsyCap, in which she envisioned helping the schools in her district to attain a high reputation like they previously had. She mentioned,

“If possible, I want to make Temerloh (state) number one again as to how it was before. Previously when I was a GC (Guru Cemerlang/Excellent Teacher), Temerloh (state) was in the first place in academic achievement in Pahang”

(Siti)

For Asyikin, her determination to help teachers was evident in her aim. According to her,

“I set as many goals as I can and one of those is to make the state that I serve successful. The aim that I set up every year is, ‘What can I do for my PPD (district office)? What can I do for my organisation? What can I do to my coachees?’ And this year, I managed to develop a module for my coachees. I even created (learning) games”

(Asyikin)

For Zaiton, her goals were no different than the other participants. It was her main priority to help teachers and students gain success in their academic affairs. She said,

“My aim is to produce quality teachers so they can further help the students to become excellent”

(Zaiton)

#### 4.3.3. Job as Calling

The participants mentioned their love and passion for their jobs. They were so passionate about the job that they were willing to invest themselves in overcoming the difficulties that came their way. Asyikin said,

“I love my job. It is because, when I enjoy what I’m doing, I will find a solution to whatever issue that comes ahead”

(Asyikin)

For Laila, she asserted, “The schedule was undeniably packed but, as Steve Job said, if we love our job, InshaAllah (with God’s permission), the job will always be fun” (Laila). She also mentioned, “I did not realise that I will soon retire because I enjoy doing my work. It’s like I can’t wait to go to work every day” (Laila). 

For Laila, having IC as her career was a true calling. Regardless of the difficulties and demanding job responsibilities, she saw it as something that made her excited every day. She did not regard the hardship she faced at work as demotivating, but rather as a challenge that she wanted to accomplish. The overall findings obtained in this study show how participants in this study experience PsyCap in their practice as instructional coaches through a feeling of responsibility for the betterment of the Malaysian education system, PsyCap as positive resources that help them brave through challenging tasks as instructional coaches and PsyCap in association to their commitment in working and pushing themselves to give the best they can to help teachers with their instructional skills.

## 5. Discussion

The participants’ experiences of PsyCap were conceptualised to three main super-ordinate themes: sense of responsibility, PsyCap as positive resources, and work commitment. The evidence from this study suggests that the participants experienced PsyCap through their feeling of responsibility toward others; realising the skills and quality in self; having a positive mind, positive attitude and behaviour; regard current job as calling, having knowledge-seeking behaviour; and setting goals in mind and working hard towards achieving those goals. As the participants developed positive psychological resources of PsyCap, it led to a range of positive outcomes that contributed to the increase in their responsibility, their commitment to work and having a positive mindset. This finding is not a mere claim, as it has been previously demonstrated through a recent meta-analytic work [50]. The findings identified in this study were similar to what has been discussed by [17,51,52,53,54]. Previous work on PsyCap has shown a positive link between PsyCap and individual behaviour in the workplace [55,56,57,58,59,60]. PsyCap has also been found to influence desirable individual behaviour, that is, individuals with a high level of PsyCap displayed an increase in job performance, managed to solve a problem at work and had improved individual well-being, as well as a range of other positive outcomes.

### 5.1. A Sense of Responsibility 

The participants’ experiences of PsyCap in the practice of instructional coaching were depicted through their sense of responsibility and the relationship they established with the teachers. They felt the need to share the knowledge they gained through years of working within various educational organisations as well as their expertise in the specific discipline. They felt it was their job to help the teachers improve their instructional skills, teach using the correct approach and to reform the current educational system. Prior to becoming ICs, these participants were lecturers and Excellent Teachers. It is safe to say that they were in their comfort zone after obtaining numerous excellent awards as well as being respected by people. However, they chose to become ICs as they felt that there was something they needed to accomplish for the betterment of the nation’s academics in the future. For them, being an IC meant being able to leverage their expertise. The participants in the study displayed their confidence in reforming the current educational system by helping teachers improve their instructional skills and, overall, flourish in their careers as teachers. The way the participants in the study believed that they had the ability to help teachers and strategised their goals toward realisation could be explained through [61,62,63] social cognitive theory. Bandura’s social cognitive theory highlights five cognitive processes that build up an individual’s self-efficacy: symbolizing, forethought, observation, self-regulation and self-reflection. The cognitive process most relevant to the participants’ efficacy was self-regulatory processing, in which they agentically set specific goals and the standard they intended to achieve as ICs. Having set specific goals made them focus on the energies in developing, improving and eventually reaching their goals. This could be seen when the participants highlighted during the interview session the goals they wanted to achieve as ICs. For instance, one participant mentioned wanting to make their teachers be on par with international level teachers, presumably teachers in developed countries. 

They did not feel that their contributions to the teachers at teacher training institutes as well as schools were enough. They wanted to accomplish more than just delivering theory on education, more than just giving talks on ways to succeed academically, and to make a bigger impact, through “hands on” practice with teachers. They believed that they could produce a much bigger impact by “stepping down” from their current employment and helping the teachers at the grassroots level, beyond just theory. They had “a positive, fulfilling, work-related state of mind characterised by vigour, dedication and absorption” [64], illustrated by high levels of energy, resilience and persistence in the face of difficulty, pride, inspiration, significance and challenges in their work [65]. When the Ministry of Education Malaysia introduced the IC position, it was as though their dream to help teachers came true. They had been waiting for this for so long as they felt that this could be the best platform to help the teachers to become better at teaching students. Having worked long enough within the educational organisation and having a tremendous amount of experience in a particular discipline made them realise that reforming the current educational system was their responsibility. This sense of responsibility toward the teachers was portrayed by the participants and paralleled what was discussed by Luthans and his colleagues, who found that when an individual experiences PsyCap, they are more responsible, resourceful and creative as well as having meaningfulness at their job [26]. In addition, when individuals experience PsyCap, they have more independence in their thinking and have a higher tendency to grow and achieve success. This can be explained through the striving component of motivation, conation. Conation facilitates several important aspects of PsyCap, that is, agency, sense of control and intentionality. It facilitates goal-directed energy, which activates motivation and the deployment of necessary resources for goal pursuit, as well as promotes positive reaction when faced with obstacles [15]. 

The participants’ experience of PsyCap was associated with feeling responsible to help others (i.e., teachers and students) to improve themselves and, furthermore, attain success. They left their previous profession as lecturers and some as Excellent Teachers to become ICs so they could help teachers. A study conducted with Filipino teachers found that PsyCap was positively linked with various psychological well-beings that included thriving at work, interpersonal fit at work, feeling competency, perceived recognition at work, job performance and desire for involvement at work [32]. This evidence supports a theme that emerged in the present study of the participants sharing their experiences of PsyCap through feeling the sense of responsibility and willingness to leap from their profession in order to help others. In addition to existing studies [17,32,51,52,53,54] another research that supported the findings of this study was conducted by [66], who investigated PsyCap concerning individual attitudes and performance. This study reported that PsyCap shows a positive link with individual attitude, specifically in job satisfaction and commitment at work [66]. It can be noted that the participants’ feelings of responsibility could very much relate to their commitment at work. They attained the highest grade within the educational organisation and were already in their comfort zone. Their willingness to venture into a newly introduced profession exemplified their positive attitude as well as their commitment to work, especially when looking at a wider perspective of them wanting to reform the country’s educational system.

### 5.2. Positive Resources 

Having the aspiration to help teachers, all the participants had goals set in mind that they wanted to achieve. And because of that, they did not easily give up when faced with challenges, be it from their coachees and administrators, the complexities of the tasks they were assigned or their expectations and goals while executing those tasks. The participants shared their experiences that embodied PsyCap as personal resources that kept them positive in challenging times while in the practice of instructional coaching. While the outcomes of instructional coaching sounded promising, it came with several challenges that the ICs had to face. They did not simply give up when meeting roadblocks in their journey of helping the teachers. It was apparent that the job of an IC was not easy as it required them to work with teachers who were reluctant to change. It requires determination and perseverance to attain success in coaching teachers. Jim Knight, who did extensive work researching instructional coaching practice, highlighted how crucial individual behaviour towards accepting instructional coaching is to the success of the practice. According to him, for coaching to succeed, one must be open to change, precisely in their instructional practice [67]. However, changing behaviour is not something that can happen overnight. Knight asserts, “Changing the way we teach requires us to change habit of behaviour, and changing habit is not easy, as anyone who has tried quit smoking, lose weight, stop spending, or increase exercising has realised”. [68] point out that personal change only occurs when someone overcomes their habitual way of living. Desire and willpower are usually insufficient to make real change occur. With regard to the transition from the previous role to the new role of IC, the participants discussed that they went through difficulties adjusting to the demanding job as an IC in the beginning. Some of the participants shared that their presence at school was not welcomed by the teachers. They were turned down and excuses were given by the teachers to avoid them. In some cases, the teachers went as far as treating them badly and using harsh words as means of resisting their presence at school. Some participants shared their experience of having a hard time convincing the teachers to receive coaching from them. They had to deal with resistance and unwelcoming teachers who refused to change their methods of teaching. The teachers openly rejected them not only through words, but also through facial expressions. It was very difficult for the ICs and some of them were really under pressure during that time. One participant mentioned her being prescribed medication by her doctor because she was diagnosed with hypertension due to the immense pressure at work. Despite what happened, she refused to give up and continued doing the best she could in her job as an IC to help the teachers. The resistance from teachers and personal attacks that the participants experienced are common among ICs [69]. 

**Figure 1:**
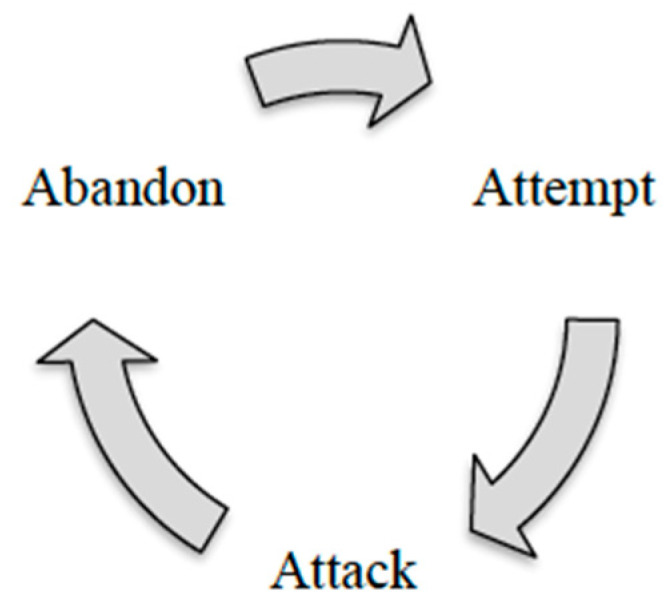
The attempt, attack, abandon Cycle.

When people are nervous about change, they feel the need to resist by voicing their resistance verbally and attacking the person who is promoting the change, in this case the ICs. Oftentimes, teachers resist change due to the vicious cycle of ”attempt, attack, and abandon” that ensures that new teaching practices are never implemented (Figure 1) [67]. This cycle has frequently occurred in schools [67,70]. During the “attempt, attack, abandon cycle”, someone introduces a new practice to a school, and at that time, teachers only make half-hearted attempts to implement it. Then, even before the practice matures and is given enough time to be fully implemented, various parties, either from schools, districts or states, begin to attack the program, hence resulting in the teachers losing their will to stick with the newly introduced practice. Furthermore, although the practice is never fully implemented and the outcomes are yet to be realised, the practice is considered unsuccessful and is abandoned by the leaders, only to propose another practice that would surely undergo the same vicious cycle [67]. Schools will continuously be drawn into this cycle, leaving teachers feeling hopeless about having a meaningful and effective change. Having served long enough in various educational organisations, the participants in the study were fully aware of this vicious cycle and it appeared that eliminating this cycle was also part of their goals.

Despite the circumstances and unfavourable experiences as well as challenges the participants experienced with teachers while in the practice of coaching, they remained positive about it despite the roadblocks they encountered. They portrayed a characteristic of an effective leader. As Fullan mentioned, “Effective leaders make people feel that even the most difficult problem can be tackled productively. They are always hopeful- conveying a sense of optimism and an attitude of never giving up in the pursuit of highly valued goals. Their enthusiasm and confidence (not certainty) are, in a word, infectious” (p. 7) [71]. They treated those challenges and difficulties as something that could help them improve themselves. For them, if they could successfully overcome those challenges, it meant that they performed their job in the best possible way. These hopeful participants were self-aware and knew their capabilities, vulnerabilities, values, emotions and goals. As highlighted by the leadership literature, when participants possess the aforementioned criteria, they are called authentic leaders [72,73,74]. The ICs, as educational leaders that support teachers towards improvement, were always positive during their coaching sessions. Research has found that positive leaders are also authentic and effective [72,74,75,76].

Seligman, a name known for his extensive research on optimism, discussed that optimistic individuals were happier, more persistent, and consequently more successful professionally and personally than those with pessimistic minds [77]. Seligman mentioned that what matters is how people think and talk to themselves during difficult times, failure and frustration. Optimists talk themselves through challenges they face and remain hopeful and energised when they encounter roadblocks. According to Seligman, “The skills of optimism do not consist in learning to say positive things to yourself. We have found over the years that the positive statements you make to yourself have little, if any, effect. What crucial is, what you think when you fail, using the power of ‘non-negative thinking’”. Changing the destructive things you said to yourself when you experience the setbacks that life deals with all of us is the central skill of optimism [77]. Inevitably, the constructs of hope, optimism, efficacy and resilience weaved together whenever challenges came in the participants’ way of meeting their goals. The integration of these constructs made the outcomes much more significant. This could be explained through Hobfoll’s conservation of resources theory [78]. It states that an individual accumulates resources to be used in times of crisis [78]. This theory emphasises the necessity to treat individuals’ psychological resources (i.e., four constructs of PsyCap) as integrated resource sets rather than as stand-alone resources. Hence, in times of crisis, these four constructs work synergistically to help the individual go through tough times, handle the situation positively and later achieve favourable outcomes. Where the participants of this study are concerned, PsyCap was the psychological resources that helped them brave through the challenging times as ICs. Because PsyCap is an asset, it can be accumulated to be utilised in the future by the individual. During tough times, the four constructs of PsyCap—hope, efficacy, resilience and optimism—were drawn upon, enabling the participants to remain positive about the situations, look for alternative ways to cope with the unfavourable situations, be confident that they could find solutions and take responsibility for the things that happened. 

Apart from resistance from the teachers, the amount of workload given to the participants was also a challenge they had to face. A lot of times, these participants were given assignments that were outside of their job scope. At times, the skills, time, and commitment required to accomplish those assignments even exceeded their capacity as an IC. However, instead of complaining, the participants took the approach of doing the best that they could. They looked for ways to solve the issue they faced. It was rooted in their personal mottos of less complaining and more doing. The passion and love that they had for their career as an IC surpassed all the challenges they encountered. They wanted to help the teachers be the best version of themselves so that they could produce excellent students. These ICs seemed to consistently draw on their personal resources in their work and create new resources for themselves through professional growth. Similarly, their investment in relationships and growth seemed to generate more PsyCap resources, increasing hope, self-efficacy, resilience and optimism [79]. Through the narratives presented by the participants in this study, they highlighted the need to be humble and honest and overall have a good personality to ensure the success of coaching with teachers. Besides being a buffer during challenging times, PsyCap has also been found to relate to individual desirable work attitudes [80,81]. On top of that, as individuals experience PsyCap, it is unlikely for them to produce undesirable attitudes at work such as cynicism, turnover intentions, job stress and anxiety, as well as counterproductive work behaviours [82]. 

### 5.3. Work Commitment

As mentioned earlier, at the beginning of their careers as ICs, the participants shared their experiences of facing several difficulties in obtaining teachers’ interest in coaching, especially from those who were reluctant. They knew they had to look for ways to obtain not only the teachers’ interest, but also trust in them. The participants realised that to make the coachees trust them, they must be knowledgeable. They knew that they had the responsibility to equip themselves with the knowledge and skills to coach people. Realising this, some of the participants in the study went the extra mile by attending courses to obtain a professional coaching certificate. They knew that they bore the responsibility to help teachers. What was more surprising was that they paid for the course using their own money. To top that off, some of them even attended tuition classes to gain a grasp of the primary school science subject. For them, understanding the subject well would make them better coaches and have the ability to coach the teachers appropriately. As the participants shared these experiences of theirs, it could be seen that they were fully committed to their job. When encountering situations that were not in their favour, they avoided complaining and instead focused on finding a solution to the issue they faced. When an individual experiences PsyCap, it increases their job satisfaction, enhances their commitment at work as well as enriches their social capital [83]. In addition, as the challenging circumstances persist, it becomes an input into a positive spiral of increased resiliency [81]. One’s level of resiliency tends to increase as they encounter challenging circumstances in several events [81,84].

## 6. Limitations and Future Research Directions

This current study has a methodological limitation in its generalisability and transferability beyond instructional coach (IC) participants due to the small sample size. There is a concrete reason to believe that generalisation could be made from the in-depth research. Merriam stated that a rich and thick description gathered from interviews performed with the participants will provide enough description so that individuals or readers beyond the population in the study would be able to determine how closely their situations match the research situation and whether the findings can be transferred [85]. Additionally, through typicality or modal category, describing how typical the individual is compared with others in the same class will enable them to make comparisons with their own situations [86]. In addition to the size of the sample, this current study only involved ICs who were responsible for coaching secondary school teachers. Teachers in secondary and primary schools might differ in terms of their level of workloads, commitment, instructional skills and approach in teaching the students, hence serving different sets of challenges. Therefore, future studies are recommended to research PsyCap with different ICs who coached teachers other than those in secondary schools. In addition, future research exploring the experience of PsyCap of instructional coaches at different position grades should be conducted. This will be treated as the follow-up study in which the sample is defined concerning the present study and this would gradually make it possible to build a bigger picture for the larger population of ICs [37]. Moreover, this will also enable theoretical generalisation, as the findings will constitute individuals from the same population. In addition, exploring PsyCap with another group of people within the academic field could also be conducted to suggest interesting findings that further add the literature concerning PsyCap from the educational organisation lens.

## 7. Conclusions

The demanding job of instructional coaches (ICs) affects them both mentally and physically and calls for an exploration to how ICs experience PsyCap so that the best possible way to develop the inner psychological resources of PsyCap that could help them to perform their job successfully can be proposed. Past research has demonstrated PsyCap to lead to several positive outcomes. It is positively related to individual attitude, specifically in one’s job satisfaction and organisational commitment [66]. Besides that, it was also found that higher PsyCap leads to more satisfaction, more commitment and high performance in doing tasks at work [28,29,51,87]. In this study specifically, the exploration of seven instructional coaches’ experiences and narratives found that PsyCap was experienced by the participants through a sense of responsibility, positive resources and work commitment. This study’s findings demonstrate the importance of having PsyCap as inner psychological resources that an instructional coach should possess. It is evident that in the 21st century, the role of IC is not restricted to only teaching teachers (i.e., coachees) the best instructional skills, but instead encompasses multifaceted areas that include managing their emotions as well as maintaining their well-being. Hence, it is pivotal for the IC, as the first line of defence, to prepare themselves by realising and developing their own inner psychological resources of PsyCap before managing others. 

To this end, our findings suggest an implication for the Ministry of Education to re-evaluate the selection criteria of ICs that would include PsyCap as one of the selection criteria. Current selection of ICs is based on the outlined criteria which are (1) Excellent Teachers/excellent lecturer; (2) have at least 5 years experience in teaching; (3) have minimum annual performance mark of 85% for 3 consecutive years; (4) have made property declaration; and (5) never undergone disciplinary actions. The criteria set for choosing ICs was mostly based on government officer grade, individual achievement and experiences. However, the guideline does not include the psychological aspect, despite its relevance and importance in carrying the challenging roles of an IC. The evidences from various studies have linked PsyCap with work performance, job attitude and behaviour [82]. Thus, including PsyCap as one of the selection criteria in appointing ICs would potentially boost the possibility for coaching success. This process can be performed using the established PsyCap self-report measure (questionnaire) called PCQ-12. The result obtained from the questionnaire, together with the existing selection criteria laid out by the ministry, may help in the process of identifying the best possible candidates for the IC position. 

In addition, the accumulated knowledge on the ICs’ PsyCap could be applied in designing appropriate and relevant PsyCap development training programmes based on the research evidence of the developable characteristics of PsyCap constructs. The programme should aim at enhancing positive psychological resources of ICs by first implicitly scrutinising factors that lead to the development of individual positive psychological resources. By doing so, future ICs will be equipped with positive psychological resources that would help prepare them for the demanding, constantly evolving and challenging task of an IC. Additionally, the PsyCap development programme could help ICs to realise their inner strengths derived from their own personal psychological resources that they may not be aware of. Therefore, the training program not only enhances individual PsyCap, but also helps ICs uncover their hidden inner positive psychological resources. 

From a theoretical viewpoint, this study contributed to the literature in several ways. The findings of the study contributed to the understanding of PsyCap from the educational organisation lens, specifically among instructional coaches, since many researches on PsyCap have been concentrated within industrial organisations, with very few researches looking into the perspective of educators and instructional coaches. As the majority of the previous data related to PsyCap were Western-based and gathered from developed countries [16], this study, being conducted in a South-East Asian country, may extend the literature to include input from other cultures, belief systems, and language backgrounds and hence provide a more inclusive data rather than just having collective evidence from western countries. This is because, since PsyCap is state-like and developmental, it could potentially be influenced by cultural context [51], which would suggest different findings from the existing ones.

## Figures and Tables

**Table 1:** Respondents’ characteristics and demographics.

Pseudonym	Age, Gender and Racial Background	Years Qualified as an Instructional IC	Previous Role
“Ahmad”	59, M, Malay	2014	Lecturer
“Laila”	57, F, Malay	2014	Lecturer
“Siti”	55, F, Malay	2014	Excellent Teacher
“Zaiton”	55, F, Malay	2013	Excellent Teacher
“Asyikin”	52, F, Malay	2014	Excellent Teacher
“Sheila”	49, F, Malay	2014	Lecturer
“Yahya”	57, M, Malay	2014	Excellent Teacher

**Table 2:** Sampling and recruitment strategy.

Prepare	Contact	Follow Up
Describing the sample: Exemplary ICs, were an instructional coach for 5 years or more, highest grade for a government officer	Initial approach: Access and ethical considerations acknowledged, all potential applicants contacted through WhatsApp to send notification of study	Feedback to and from participants: Reiteration of participant information provided
Finding information sources: Determine potential group for target sample	Negotiation with key contacts: Negotiation with School Management Division and Educational Planning and Research Division, State Education Department. Request for ethical permission from University Ethics Committee for Research Involving Human Subjects (JKEUPM)	Feedback from key contacts: Findings discussed with research team
Discovering recent or related projects: No other research studies involving target sample at time of study	Direct negotiations: Requests made for potential participants. Information sheet sent out to all ICs with assurance of anonymity/confidentiality	Continuing links: Agreement received from individual participants to provide face-to-face feedback

**Table 3:** Super-ordinate and sub-ordinate themes of ways participants experienced PsyCap.

Super-Ordinate Themes	Sub-Ordinate Themes
A sense of responsibility	Responsibility to others Realising the skills and quality in self
Positive resources	Keeping a positive mind Positive attitude and behaviour
Work commitment	Having goals set in mind and working hard towards achieving those goals Knowledge-seeking behaviour Job as calling

## Data Availability

The data presented in this study are available on request from the corresponding author.
